# Mitophagy-driven immune evasion in skin cancer: multidimensional regulatory networks and context-dependent therapeutic strategies—a narrative review

**DOI:** 10.3389/fimmu.2026.1949371

**Published:** 2026-09-07

**Authors:** Jian Lv, Guijun Liu

**Affiliations:** 1 Graduate School, Heilongjiang University of Chinese Medicine, Harbin, China; 2 Department of Dermatology, The Fourth Affiliated Hospital of Heilongjiang University of Chinese Medicine, Harbin, China

**Keywords:** context-dependent therapy, immune evasion, mitophagy, PD-L1, skin cancer, tumor microenvironment

## Abstract

Mitophagy serves as a pivotal metabolic-immune hub that drives immune evasion in major skin cancer subtypes including melanoma cutaneous squamous cell carcinoma and basal cell carcinoma. It acts as a core barrier to effective immunotherapy. Mitophagy activates canonical mitophagic pathways such as PINK1 Parkin and BNIP3 NIX. It extensively remodels the tumor immune microenvironment through multiple mechanisms. It skews tumor-associated macrophages toward immunosuppressive M2 polarization. It impairs antigen presentation of epidermal Langerhans cells. It induces exhaustion of CD8^+^ cytotoxic T lymphocytes. It also modulates pro-inflammatory cytokines including IL-1β and TNF-α. Mitophagy triggers sequential activation of NF-κB NLRP3 inflammasome and cGAS-STING signaling at the same time. It couples metabolic reprogramming and epigenetic modifications to sustain immunosuppression. A skin-cancer-specific UV-microphthalmia-associated transcription factor (MITF) regulatory axis mediates the crosstalk between mitophagy and PD-L1 expression. It generates heterogeneous and subtype-specific immune microenvironments. It further promotes resistance to immune checkpoint blockade. Mitophagy also modulates tumor antigen presentation immune checkpoint profiles and metabolic fitness of infiltrating immune cells to amplify immune evasion. This narrative review systematically delineates the multidimensional regulatory networks of mitophagy-driven immune evasion in skin cancer. It clarifies context-dependent regulatory patterns across distinct histological subtypes. It proposes precision-targeted therapeutic strategies by combining mitophagy modulation with immunotherapy. These insights provide novel mechanistic frameworks and translational targets to overcome immunotherapy resistance. They also help improve clinical outcomes for patients with advanced skin cancer.

## 1 Introduction

Skin cancer mainly comprises three major subtypes, including melanoma, squamous cell carcinoma (SCC) and basal cell carcinoma (BCC). Melanoma exhibits the highest degree of malignancy, and the five-year survival rate of patients with metastatic melanoma is extremely low (1, 2). SCC has the highest incidence among all types of skin cancer. It is prone to regional lymph node metastasis and carries a high risk of recurrence after treatment (3, 4). BCC shows relatively weak invasiveness. However, long-term ultraviolet exposure can induce multiple lesions that are difficult to achieve radical cure (5, 6). The clinical application of immune checkpoint inhibitors has completely transformed the therapeutic landscape of advanced skin cancer. The therapeutic efficacy of these agents has been significantly improved, especially in the treatment of melanoma (7, 8). Nevertheless, clinical data indicate that more than half of patients with advanced melanoma will develop resistance to immunotherapy. The efficacy of immune-checkpoint inhibitors in SCCAs is low (9). BCC is regarded as an immune-cold tumor, so immunotherapy can barely exert its therapeutic effect (10, 11). Tumor immune evasion is the core cause of immunotherapy resistance. Elucidating the molecular mechanisms of immune evasion in skin cancer is a key direction to break through the bottlenecks of clinical treatment.

Mitochondrial autophagy, also known as mitophagy, is a selective form of autophagy that clears damaged mitochondria. It plays an essential role in maintaining cellular mitochondrial homeostasis and energy metabolic balance (12, 13). Mitophagy shows a dual regulatory role in the process of tumor occurrence and development. It can inhibit carcinogenesis by eliminating damaged mitochondria at the early stage. At the late stage, it is hijacked by tumor cells to promote tumor cell survival and drug resistance by sustaining mitochondrial function (14–16). Recent studies have verified that mitophagy is involved in tumor immune evasion. It regulates the functions of immune cells in the tumor microenvironment, the activation of inflammatory signals, and the expression of immune checkpoints to achieve this effect (7, 12, 17). Abnormal regulation of mitophagy disrupts immune homeostasis in the tumor microenvironment. It drives the formation of an immunosuppressive microenvironment and further reduces the response rate to immunotherapy (8, 18, 19).

Recent studies have confirmed that mitophagy participates in the regulation of immune checkpoint expression in tumors. This process is mediated by modulating several critical signaling axes, including the cGAS-STING, JAK-STAT, NF-κB, and HIF-1α/PD-L1 pathways. The bidirectional regulation between mitophagy and PD-L1 in skin cancer also shows unique regulatory features that are mediated by ultraviolet radiation and microphthalmia-associated transcription factor (MITF) (20–22). The core regulatory role of mitophagy in tumor immune evasion has been widely documented. However, there is still a lack of systematic integration and in-depth analysis regarding subtype-specific mitophagy regulatory mechanisms, immune cell lineage-specific effects, and context-dependent therapeutic strategies in skin cancer. This gap has become a major obstacle for the precise intervention and clinical translation of mitophagy-targeted therapeutic approaches (14, 15, 19).

This review systematically summarizes the core ubiquitin-dependent and ubiquitin-independent regulatory pathways of mitophagy in skin cancer. It elaborates on the multidimensional molecular mechanisms through which mitophagy drives immune evasion in skin cancer. The article further analyzes the heterogeneous mitophagic features and immune phenotypes across melanoma, BCC and SCC. It places a specific focus on the bidirectional regulatory network between mitophagy and PD-L1. Context-dependent mitophagy-targeted therapeutic strategies are also proposed for skin cancer classified by distinct immune subtypes. This work aims to define the central role of mitophagy as a metabolic-immune regulatory hub in skin cancer. It provides systematic theoretical support and translational directions for overcoming immunotherapy resistance in skin cancer and developing next-generation precise combined immunotherapy regimens.

## 2 Core regulatory mechanisms of mitophagy in skin cancer

### 2.1 Ubiquitin-dependent mitophagy pathway

**Figure 1:**
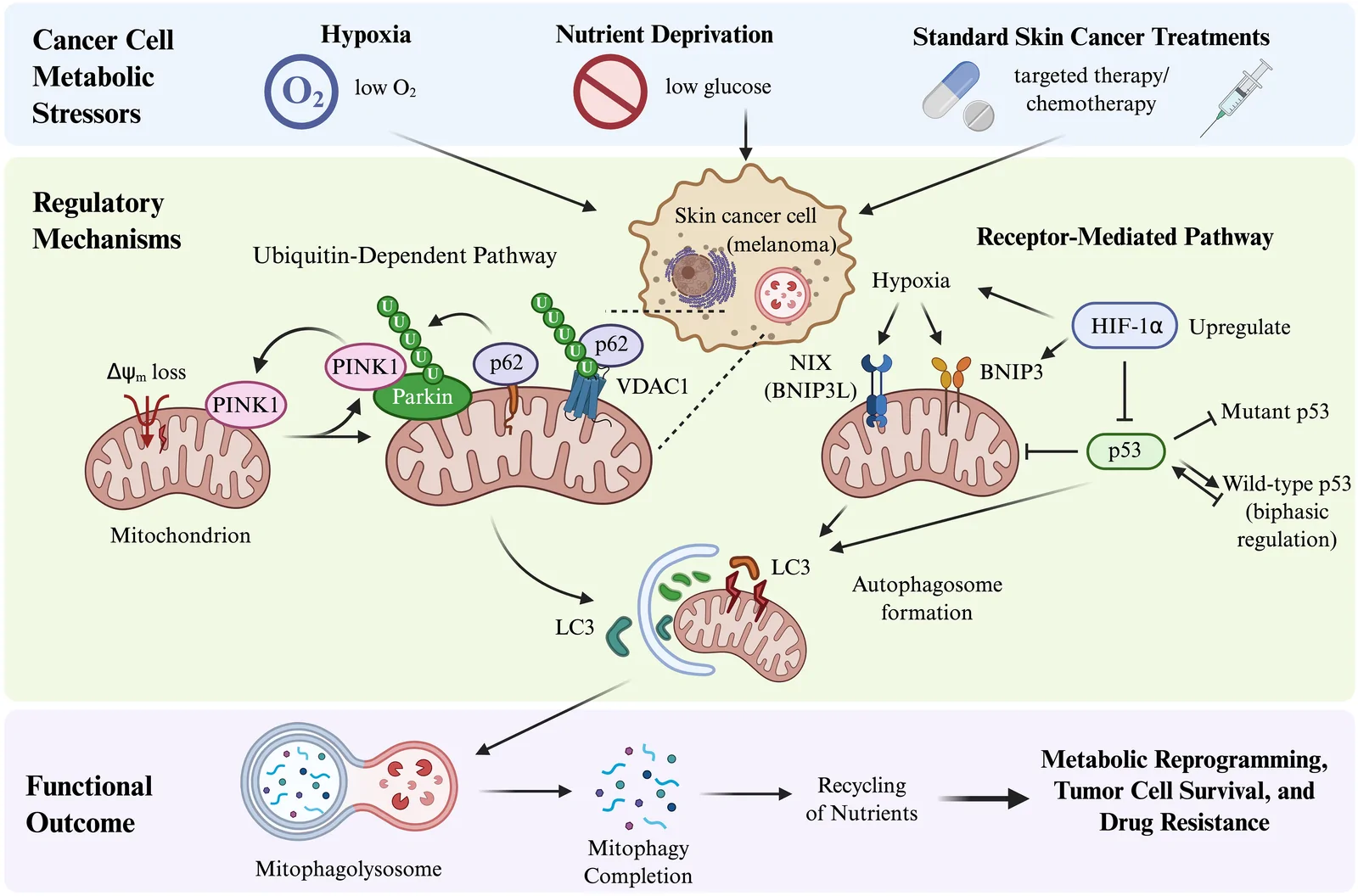
Core regulatory mechanisms of mitophagy in skin cancer. The schematic illustrates the multidimensional regulatory network of mitochondrial autophagy (mitophagy) that drives tumor progression and drug resistance in skin cancer. Mitophagy is a selective form of autophagy that clears damaged mitochondria to maintain cellular mitochondrial homeostasis and energy metabolic balance. Cancer Cell Metabolic Stressors (Triggers): The process is initiated by various microenvironmental and therapeutic stressors common in skin cancer, including hypoxia, nutrient deprivation (e.g., low glucose or glutamine), and standard treatments like chemotherapy or targeted therapies. Exposure to ultraviolet (UV) radiation also triggers mitochondrial damage and activates mitophagy to facilitate cellular adaptation. Regulatory Mechanisms: Ubiquitin-Dependent Pathway: This pathway is centered on the PINK1-Parkin axis. When mitochondrial membrane potential (Δψm) decreases, PINK1 accumulates on the outer mitochondrial membrane and phosphorylates Parkin, inducing its activation. Activated Parkin mediates the ubiquitination of mitochondrial surface proteins, which further recruits autophagy receptors. Receptor-Mediated (Ubiquitin-Independent) Pathway: In the hypoxic tumor microenvironment, HIF-1α transcriptionally upregulates the expression of receptors such as BNIP3 and NIX (BNIP3L). These receptors directly bind to LC3 to initiate mitophagy without relying on Parkin-mediated ubiquitination. Mitophagy Modulation: Signaling nodes such as HIF-1α and p53 serve as critical regulators. While HIF-1α amplifies mitophagic signals, p53 exhibits a complex regulatory role; wild-type p53 can exert biphasic regulation, whereas mutant forms may inhibit protective mitophagy. Functional Outcomes: Degradation and Recycling: Convergence of these pathways leads to autophagosome formation around damaged mitochondria and subsequent fusion with a lysosome to form a mitophagolysosome.

The core regulatory mechanism of mitochondrial autophagy in skin cancer is shown in **Figure 1**. Ubiquitin-dependent mitophagy is centered on the PINK1-Parkin axis, which represents the most prevalent regulatory pattern of mitophagy in skin cancer. Under physiological conditions with intact mitochondrial membrane potential (Δψm), PINK1 is continuously imported into the inner mitochondrial membrane and cleaved by the PARL protease, leading to its rapid proteasomal degradation. When Δψm dissipates upon mitochondrial damage, PINK1 stabilizes and accumulates on the outer mitochondrial membrane (OMM) (23). Accumulated PINK1 phosphorylates both ubiquitin chains on OMM proteins and the ubiquitin-like domain of Parkin at Ser65 (24). This dual phosphorylation unlocks the autoinhibited conformation of Parkin and activates its E3 ubiquitin ligase activity. Activated Parkin further catalyzes K48- and K63-linked ubiquitination of multiple OMM substrates, including mitofusin 1/2, voltage-dependent anion channel 1 and mitochondrial Rho GTPase 1. These ubiquitinated mitochondrial proteins are subsequently recognized by autophagy adaptor proteins such as optineurin, calcium-binding and coiled-coil domain 2 and SQSTM1/p62, which bind to LC3 via their LC3-interacting region motifs to mediate autophagosome biogenesis and enclosure of damaged mitochondria (25).

In cancer, expression levels of PINK1 and Parkin are significantly increased in melanoma. The overactivated PINK1-Parkin pathway continuously clears damaged mitochondria to sustain mitochondrial homeostasis and redox balance in tumor cells (26). High expression of PINK1 is directly associated with chemoresistance in squamous cell carcinoma. Inhibition of PINK1 expression restores the sensitivity of tumor cells to chemotherapeutic drugs by blocking damaged mitochondria clearance and accumulating pro-apoptotic mitochondrial reactive oxygen species (27, 28). Ultraviolet exposure activates the PINK1-Parkin pathway in basal cell carcinoma. Mitophagy mediated by this pathway eliminates mitochondria injured by ultraviolet radiation, which supports tumor cells in evading apoptosis (29).

### 2.2 Ubiquitin-independent mitophagy pathway

Ubiquitin-independent mitophagy does not depend on Parkin-mediated ubiquitination. This pathway mainly completes mitophagy through direct binding between mitochondrial membrane receptors and LC3. These receptors include BNIP3, NIX, FUN14 domain containing 1 (FUNDC1) and Optineurin (OPTN) (18, 30–32). Hypoxia acts as the core upstream activator of this pathway. In the hypoxic tumor microenvironment of skin cancer, hypoxia-inducible factor 1α transcriptionally upregulates the expression of BNIP3 and NIX, which directly bind to LC3 to initiate mitophagy without requiring Parkin-mediated ubiquitination (30, 33). In melanoma, BNIP3 not only mediates hypoxia-induced mitophagy but also forms a positive regulatory loop with HIF-1α via iron metabolism modulation. BNIP3-mediated mitophagy inhibits nuclear receptor coactivator 4-dependent ferritinophagy, reduces intracellular labile iron levels, and thereby suppresses prolyl hydroxylase 2-mediated HIF-1α degradation. This feedforward loop amplifies mitophagy signals and promotes tumor proliferation and metastasis (33, 34). Hypoxia-induced BNIP3 mediates mitophagy in uveal melanoma, which reprograms tumor cell metabolism toward enhanced oxidative phosphorylation and strengthens the invasion and metastasis abilities of tumor cells (30). As a hypoxia-sensitive mitophagy receptor, FUNDC1 shows high expression in melanoma. It can drive immune evasion by inhibiting anti-tumor immune responses (18). OPTN functions as a critical autophagy receptor. It is closely associated with immune cell infiltration in melanoma and acts as an important molecule for regulating the tumor immune microenvironment (17).

### 2.3 Unique drivers of mitophagy in skin cancer

Ultraviolet radiation exposure stands as the predominant environmental inducer of skin cancer. Ultraviolet irradiation triggers excessive generation of mitochondrial reactive oxygen species. This process leads to mitochondrial damage and subsequently activates mitophagy (22, 35, 36). Simulated solar radiation can induce mitochondrial fragmentation in melanoma cells. It activates mitophagy to facilitate adaptation to the oxidative stress environment. This process contributes to the development and progression of sunlight-induced melanoma (35). MITF acts as a melanocyte-specific transcription factor. It can inhibit mitophagy by regulating long non-coding RNAs including LENT. This regulation helps maintain the survival and proliferation of melanoma cells (20). Ceramide signaling serves as a vital regulatory pathway for mitophagy in skin cancer. Senescence-related ceramide signaling can induce excessive mitophagy in T cells. This effect suppresses anti-tumor immune responses (37). In head and neck SCC, ceramide analogs can activate Parkin-mediated mitophagy. This activation is realized through the regulation of DRP1 nitrosylation and fumarate metabolism (38). Nutritional stress, such as glutamine deprivation, can trigger mitophagy in SCC cells. It modifies the composition of mitochondrial RNAs in extracellular vesicles. These alterations participate in intercellular communication within the tumor microenvironment (39). In addition, disrupted metal homeostasis, abnormal ceramide metabolism and human papillomavirus infection in the cutaneous tumor microenvironment all function as specific driving factors of mitophagy in skin cancer (38, 40, 41). The core regulatory mechanism of mitochondrial autophagy in skin cancer is shown in **Figure 1**.

## 3 Multidimensional regulation of skin cancer immune evasion driven by mitophagy

### 3.1 Reprogramming of immune cell functions

**Figure 2:**
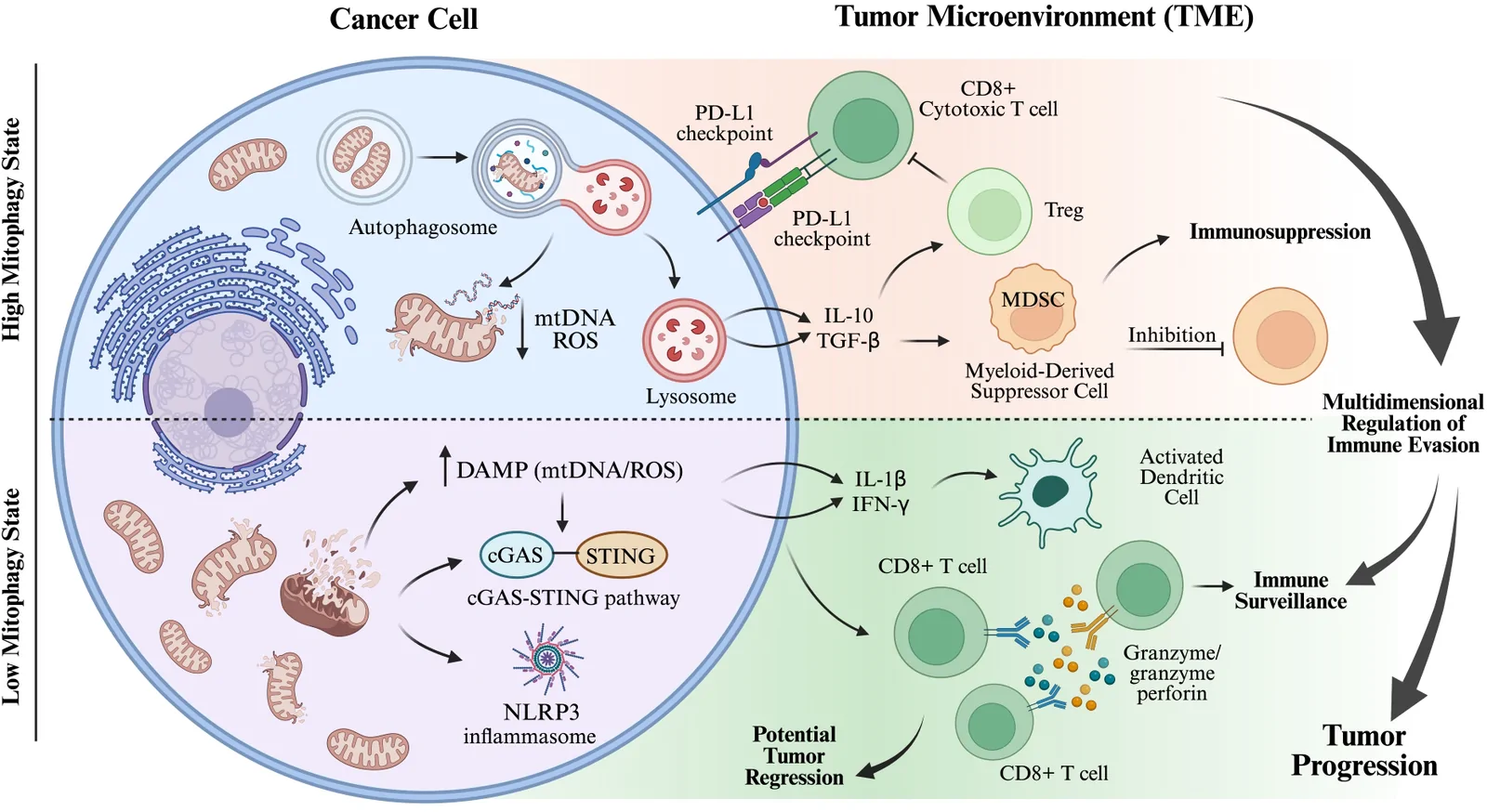
Multidimensional regulation of skin cancer immune evasion driven by mitophagy. This schematic delineates the dual role of mitophagy in modulating the skin cancer immune microenvironment, highlighting how mitochondrial homeostasis serves as a metabolic-immune hub for immune evasion. The diagram contrasts the intracellular signaling and resulting tumor microenvironment (TME) phenotypes between high and low mitophagy states. High Mitophagy State (Intracellular Mechanisms): Overactivated mitophagy ensures the efficient clearance of damaged mitochondria through the formation of autophagosomes and their subsequent fusion with lysosomes. This process significantly reduces the cytosolic release of mitochondrial reactive oxygen species (ROS) and mitochondrial DNA (mtDNA). The sequestration of these damage-associated molecular patterns (DAMPs) suppresses the activation of the cGAS-STING and NLRP3 inflammasome pathways. Immunosuppressive Tumor Microenvironment (TME): The high mitophagy state promotes an immunosuppressive profile characterized by the increased secretion of cytokines such as IL-10 and TGF-β.These factors facilitate the recruitment and activation of Myeloid-Derived Suppressor Cells (MDSCs) and regulatory T cells (Tregs), which actively inhibit the cytotoxic function of CD8^+^ T lymphocytes. Furthermore, mitochondrial homeostasis helps maintain high surface expression of the PD-L1 checkpoint, which binds to PD-1 on T cells to induce functional exhaustion. Collectively, these multidimensional regulatory networks culminate in robust immune evasion and accelerated tumor progression. Low Mitophagy State (Intracellular Mechanisms): Defective or impaired mitophagy leads to the massive accumulation of damaged, fragmented mitochondria within the cancer cell. The resulting leakage of mtDNA and ROS into the cytosol triggers the sequential activation of the cGAS-STING signaling axis and the NLRP3 inflammasome. Pro-inflammatory TME and Immune Surveillance: Activation of these inflammatory pathways induces the release of pro-inflammatory cytokines, including IL-1 and IFN-γ. In this context, the TME shifts toward an “immune-hot” phenotype, promoting the activation of dendritic cells and the infiltration of CD8^+^ T cells. Activated cytotoxic T cells exert anti-tumor effects through the release of granzymes and perforin, potentially leading to immune-mediated tumor regression.

Mitophagy promotes the development of immune evasion in skin cancer (**Figure 2**). It acts by reshaping the functional status of various immune cells in the tumor microenvironment. We have compiled relevant studies on specific mitochondrial autophagy in immune cell-mediated anti skin cancer responses in recent years (**Table 1**). Tumor-associated macrophages (TAM) are the most abundant immune cells within the tumor microenvironment. Abnormally activated mitophagy in melanoma can drive the polarization of TAM toward an M2-like immunosuppressive phenotype. These cells release anti-inflammatory factors and suppress the infiltration and cytotoxic activity of CD8^+^ T cells (8, 42). Langerhans cells represent a unique dendritic cell subset in cutaneous tissue. Impaired mitophagy reduces the antigen-presenting ability of Langerhans cells. These cells can no longer effectively initiate anti-tumor immune responses and thus disrupt local immune surveillance in the skin (15, 43). T cells act as the core effector cells of anti-tumor immunity. Melanoma cells can transfer mitochondria containing mutated mitochondrial DNA to T cells. This process inhibits mitophagy in T cells and induces their senescence and functional exhaustion (7). Senescence-related ceramide signaling triggers excessive mitophagy in CD8^+^ T cells. This leads to mitochondrial depletion and disordered energy metabolism. The affected CD8^+^ T cells eventually lose their anti-tumor effector functions (37). The proliferation and activation of regulatory T cells are also modulated by mitophagy. Overactivated mitophagy in the skin cancer microenvironment can strengthen the immunosuppressive function of regulatory T cells (37, 44). In the SCC microenvironment, mitophagy promotes the apoptosis of dendritic cells. This weakens the efficiency of antigen presentation and interrupts the initiation of anti-tumor immune responses (45).

**Table 1:** Context-specific mitophagy in immune cell-mediated anti-skin cancer responses.

Immune cell	Mitophagy status	Mitochondrial outcome	Cell phenotype	Immunological consequence	Reference
LCs, (skin-specific DCs)	Impaired/defective	Damaged mitochondria accumulation, mtROS overproduction	Antigen-presenting dysfunction, immature state	Failed to initiate anti-tumor T cell response, local immune surveillance disruption	(62, 96)
CD8^+^ T cells	Excessive activation/inhibited	Mitochondrial depletion/damaged mitochondria accumulation	Senescence, exhaustion, metabolic dysfunction	Loss of cytotoxic effector function, impaired anti-tumor immunity	(7, 48)
Tregs	Overactivated	Enhanced mitochondrial fitness, metabolic stability	Increased proliferation, enhanced immunosuppressive function	Strengthened immune suppression, promoted tumor immune escape	(37, 44)
TAMs	Overactivated	Metabolic reprogramming, reduced mtROS	M2-like immunosuppressive polarization	Inhibited CD8^+^ T cell infiltration, secreted anti-inflammatory factors	(42, 111)
Neutrophils	Inhibited (downregulated BNIP3)	Mitochondrial fission enhancement, dysfunction	Apoptosis, reduced infiltration	Impaired anti-tumor immunity, immunotherapy resistance	(45)
Th17 cells	Limited/attenuated	Sustained mitochondrial integrity, high OXPHOS	Apoptosis-resistant, long-lived persistent phenotype	Enhanced cellular persistence in TME, promoted immune dysregulation	(112)

DCs, Dendritic cells; Tregs, Regulatory T cells; TAMs, Tumor-associated macrophages; LCs, Langerhans cells; OXPHOS, Oxidative Phosphorylation.

### 3.2 Coupling between metabolic reprogramming and immunosuppression

Mitophagy contributes to the coupling of metabolic reprogramming and immunosuppression in skin cancer cells. This interaction further enhances tumor immune evasion. Mitophagy can reinforce the Warburg effect in Oral squamous cell carcinoma (OSCC). It eliminates functionally intact mitochondria and drives tumor cells to rely mainly on glycolysis for energy production (46). This process reduces nutrient availability for immune cells in the tumor microenvironment. It also leads to massive lactate accumulation and generates an acidic immunosuppressive microenvironment (47). Remodeling of lipid metabolism represents another important coupling mechanism. Succinate serves as an intermediate metabolite in the tricarboxylic acid cycle. It can maintain the stemness of CD8^+^ T cells through BNIP3-mediated mitophagy. Skin cancer cells may competitively consume succinate and disrupt the metabolic homeostasis of T cells (48). Hypoxia-induced mitophagy reprograms cellular metabolism in melanoma. It enhances oxidative phosphorylation and reduces mitochondrial reactive oxygen species production. These changes sustain metabolic homeostasis in tumor cells and simultaneously suppress anti-tumor immunity (30). Mitophagy is activated in cancer-associated fibroblasts within the SCC microenvironment. These activated fibroblasts secrete lactate to promote glycolysis in tumor cells. This forms a metabolically symbiotic microenvironment that further impairs immune cell functions (46, 49). In addition, mitophagy-mediated glutamine metabolic reprogramming induces amino acid imbalance in the tumor microenvironment. This imbalance aggravates functional impairment in immune cells (39). Mitochondria-targeted agents can reverse metabolic reprogramming and immunosuppression in OSCC cells by regulating mitophagy. They restore the anti-tumor functions of immune cells (49). Transplantation of functional healthy mitochondria into melanoma cells triggers lethal mitophagy, a pathological excessive mitophagic flux distinct from homeostatic protective mitophagy. Lethal mitophagy arises from sustained overactivation of Parkin-dependent mitochondrial clearance driven by the influx of intact foreign mitochondria, leading to global depletion of endogenous damaged organelles, collapse of tumor energy metabolism and subsequent tumor cell death (50–52). Unlike basal protective mitophagy that selectively removes fragmented mitochondria to maintain tumor survival, this mitochondria-transplant-induced lethal mitophagy disrupts tumor metabolic reprogramming, corrects glycolytic abnormalities in melanoma and breaks the coupling between metabolic disorders and immunosuppression (53).

### 3.3 Epigenetic modification-mediated stabilization of immunosuppressive phenotypes

Mitophagy can stabilize the immunosuppressive phenotype of skin cancer cells through epigenetic modifications. This process further consolidates the immune evasion state of tumor cells. The metabolite fumarate regulates the succinylation modification of Parkin. Such regulation changes the level of mitophagy and ultimately stabilizes the immunosuppressive epigenetic features in melanoma (38, 48). Noncoding RNAs also play critical regulatory roles. MicroRNAs including miR-1-3p and miR-361-5p can target and modulate mitophagy-associated genes. These events further influence the immune phenotype of skin cancer (54–56). The long noncoding RNA TEX41 regulates mitophagy signaling pathways. It reshapes the pattern of immune cell infiltration in cutaneous melanoma and serves as an important molecule in epigenetic regulation (57). Long noncoding RNAs and microRNAs in melanoma can target key mitophagy mediators. They regulate the immune phenotype of tumor cells and facilitate tumor immune evasion (20, 58). The MITF-regulated long noncoding RNA LENT suppresses mitophagy. This inhibition maintains the stemness of melanoma cells and sustains their epigenetic phenotype linked to immune evasion (20). In SCC, microRNAs directly target mitophagy-related genes. They control the epigenetic modification status of tumor cells and affect the expression of immune checkpoint proteins (54, 59). In HPV-associated SCC, autophagy-related epigenetic modifications selectively regulate mitophagy. These modifications strengthen the immune evasion traits of tumor cells (40, 60).

## 4 Bidirectional regulatory network between mitophagy and PD-L1 in skin cancer

### 4.1 Overactivated mitophagy drives PD-L1 upregulation

Excessive activation of mitophagy elevates PD-L1 expression in skin cancer cells through multiple signaling pathways. This process mediates tumor immune evasion. Overactivated mitophagy facilitates the clearance of damaged mitochondria. It reduces the release of mitochondrial reactive oxygen species and mitochondrial DNA. These changes suppress the cGAS-STING pathway and the secretion of type I interferons. Such events indirectly lead to the upregulation of PD-L1 expression (61). Mitophagy can also stabilize the expression of HIF-1α and STAT3. This stabilization directly triggers the transcriptional activation of PD-L1. The elevated PD-L1 level further restrains the anti-tumor function of T cells (44, 62). In melanoma, FUNDC1- and PINK1-mediated mitophagy maintains mitochondrial homeostasis. It reduces the release of damage-associated molecular patterns. This sequence of events upregulates PD-L1 expression and weakens anti-tumor immune responses (18, 63). In SCC, ULK1 deficiency combined with p53 activation induces PD-L1 upregulation. This induction relies on reactive oxygen species and the NLRP3 pathway and strengthens the immunosuppressive state (64). Overactivated mitophagy also stimulates HIF-1α signaling. These pathways transcriptionally upregulate PD-L1 expression and suppress T cell functions (8, 33).

### 4.2 PD-L1 induction mediated indirectly by impaired mitophagy

Defective mitophagy gives rise to massive accumulation of damaged mitochondria. The released mitochondrial DNA activates downstream inflammatory signaling and further induces PD-L1 expression. In melanoma, impaired mitophagy caused by loss of Parkin function aggravates mitochondrial damage. This damage activates the cGAS-STING-TBK1 pathway and promotes transcriptional upregulation of PD-L1. Such changes reduce the therapeutic efficacy of immune checkpoint inhibitors (61, 65). In SCC, mitophagy deficiency resulting from low PINK1 expression activates PD-L1 transcription through inflammatory signaling pathways. This mechanism mediates resistance to immunotherapy (66, 67). In BCC, ultraviolet-induced mitophagy impairment triggers chronic inflammatory responses. These responses act in synergy to induce high PD-L1 expression and establish an immunosuppressive microenvironment (22, 68).

### 4.3 Unique regulatory characteristics in skin cancer

Skin cancer harbors a specific closed regulatory loop involving ultraviolet radiation, MITF, mitophagy and PD-L1. This regulatory pattern is not found in other tumor types. Ultraviolet exposure modulates mitophagy levels in melanoma through the MITF axis. Such modulation further affects the expression of PD-L1 (20, 22). As a melanocyte-specific transcription factor, MITF can directly regulate the expression of mitophagy-related genes. It also indirectly controls the transcriptional level of PD-L1 through mitophagy. A bidirectional regulatory loop is therefore established (20). In head and neck SCC, the expression signature of mitophagy-related genes shows a positive correlation with PD-L1 expression level. This signature can be used as a predictive biomarker for immunotherapy response (8, 69). The regulation of PD-L1 by mitophagy in skin cancer cells is context-dependent. Protective mitophagy and lethal mitophagy exert completely opposite effects on PD-L1 expression. This distinction also serves as a key contributor to immunotherapy heterogeneity in skin cancer (14, 16).

## 5 Subtype heterogeneity and mitophagy characteristics in skin cancer

### 5.1 Melanoma

The characteristics of mitochondrial autophagy and related immune dysregulation in different subtypes of skin cancer are shown in **Table 2**. Melanoma is recognized as an immune-hot tumor. It displays immune hallmarks featured by high T-cell infiltration and positive PD-L1 expression. Mitophagy in melanoma is mainly characterized by excessive activation. Moreover, differences in core mitophagy pathways exist across gene mutation subtypes (14, 19, 70). BRAF-mutant melanoma mainly depends on the PINK1-Parkin ubiquitin-dependent pathway. Overactivated mitophagy maintains mitochondrial homeostasis in tumor cells. It also drives the polarization of tumor-associated macrophages into an immunosuppressive phenotype and induces exhaustion of CD8^+^ T cells (70, 71). NRAS-mutant melanoma primarily relies on the BNIP3-NIX ubiquitin-independent pathway. Hypoxia-triggered mitophagy reprograms tumor metabolism and facilitates tumor invasion and metastasis (30, 33). Metastatic melanoma can suppress mitophagy in T cells via mitochondrial transfer. This event further reinforces immune evasion and acts as a critical mechanism for immunotherapy resistance in advanced melanoma (7).

**Table 2:** Characteristics of mitochondrial autophagy and related immune dysregulation in different subtypes of skin cancer.

Tumor type	Cancer type	Mitophagy status	Activation conditions	Signaling pathway	Key molecular targets	Result	Reference
Immune-hot	Melanoma	Overactivated	ER stress, mitochondrial fission	XBP1-MARCH5-MFN2	MFN2, DRP1	Confers ER stress resistance and immune escape	(107)
		Defective	Parkin inactivation, mtDNA accumulation	cGAS-STING-TBK1	IFN, IL-6	Induces PD-L1 expression and immunotherapy resistance	(65)
		Overactivated	UV exposure, BRAF mutation, mitochondrial stress	PINK1-Parkin	PINK1, Parkin	In the presence of 1,25(OH)2D3, glycolysis is enhanced along with energy-conserving processes such as autophagy and mitophagy, resulting in increased repair of cyclobutane pyrimidine dimers and decreased oxidative DNA damage.	(36)
			Hypoxia, NRAS mutation	HIF-1α/BNIP3	BNIP3, NCOA4, PHD2	Promotes metabolic reprogramming and tumor metastasis	(34)
			Oxidative stress, mitochondrial damage	PINK1-Parkin	MMP-2, MMP-9,	Elevated ROS levels induce mitochondrial dysfunction	(113)
			MITF dysregulation, UV radiation	MITF/LENT	MITF, LENT	Maintains melanoma stemness and suppresses anti-tumor immunity	(20)
			Hypoxia, iron metabolism disorder	HIF-1α/BNIP3	BNIP3, HIF-1α, NCOA4	Stabilizes HIF-1α and accelerates tumor growth	(33)
			Mitochondrial transfer from cancer cells	ROS/PINK1-Parkin	PINK1-Parkin	Reduced the ratio of M2 macrophage in tumor microenvironment and cell metabolism	(23)
			Ceramide signaling, aging stress	Ceramide/DRP1	Parkin, fumarate	Ceramide-mediated mitophagy and tumor suppression are regulated by Drp1 nitrosylation, fumarate depletion, and PARKIN succination	(38)
Immune-cold	Basal cell carcinoma	Moderately activated	Hedgehog pathway overactivation	Hedgehog	SRF, GLi1, MKL1	Reduces tumor cell viability	(28)
		Defective	stress, mitochondrial damage	PINK1-Parkin	MFN-1, DRP-1, OPA1	Disrupting mitochondrial dynamics via ROS	(68)
Moderate immune infiltration	Oral squamous cell carcinoma	Overactivated	Hypoxia	BNIP3	BNIP3	Promotes cancer stem cell stemness and invasion	(79)
	Esophageal squamous cell carcinoma	Overactivated	mitochondrial damage	PINK1-Parkin	LC3, ROS, Parkin	Inhibition of autophagy and mitophagy	(67)
	Head and neck squamous cell carcinoma	Overactivated	TKI resistance, redox imbalance	SOD2/PINK1-Parkin	SOD2, mtROS	Reduces TKI sensitivity and maintains mitochondrial homeostasis	(105)
	Uveal melanoma	Overactivated	Hypoxia, mitochondrial dysfunction	BNIP3	OXPHOS, HIF-1α	Drives metastatic progression and immune suppression	(30)
	Tongue squamous cell carcinoma	Overactivated	CAFs-derived lactate, miR-26a-5p downregulation	PI3K/Akt/mTOR	Parkin, miR-26a-5p	Accelerates tumor proliferation and migration	(114)

BNIP3, Bcl-2/adenovirus E1B 19kDa interacting protein 3; CAS, cyclic gmp-amp synthase; CAFs, cancer-associated fibroblasts; NCOA4, nuclear receptor activator 4; PHD2, prolyl hydroxylase 2; UV, ultraviolet; SRF, serum response factor; MKL1, megakaryoblastic leukemia 1; GLI1, glioma-associated oncogene family zinc finger-1; MFN-1, mitofusin-1; DRP1,dynamin-related Protein 1; OPA1,optic atrophy 1; TKI, tyrosine kinase inhibitor; PINK, PTEN-induced putative kinase 1; SOD2, Superoxide Dismutase 2; MMP-2, Matrix Metalloproteinase 2; MMP-9, Matrix Metalloproteinase 9; MITF, microphthalmia-associated transcription factor.

### 5.2 Basal cell carcinoma

BCC is recognized as an immune-cold tumor. It shows immune features characterized by low T-cell infiltration and a reduced number of Langerhans cells (72). Mitophagy in BCC is mainly marked by moderate activation (68). The occurrence of BCC is closely linked to long-term ultraviolet exposure. Ultraviolet-induced moderate mitophagy clears damaged mitochondria. It suppresses immune cell recruitment mediated by the cGAS-STING pathway and maintains an immune-desert state (73, 74). A synergistic regulatory interaction exists between mitophagy and the Hedgehog pathway in BCC. Abnormally activated Hedgehog signaling upregulates mitophagy activity. This change further weakens local immune surveillance (75, 76). Dysregulated mitophagy in Langerhans cells serves as the central reason for the immune-cold phenotype of BCC. Impaired mitophagy results in the loss of antigen-presenting function in Langerhans cells. These cells then fail to launch effective anti-tumor immune responses (77, 78).

### 5.3 Squamous cell carcinoma

SCC is categorized as a tumor with moderate immune infiltration. Mitophagy in this malignancy exhibits features of heterogeneity (60, 79). Mitophagy is excessively activated in hypoxic regions at the tumor core. Mitophagy is impaired in peripheral tumor regions and gives rise to local inflammatory responses (23). Significant differences in mitophagy profiles are observed between HPV-associated and non-HPV-associated SCC. The HPV E7 oncoprotein inhibits general autophagy by degrading AMBRA1. It also selectively promotes mitophagy to reinforce immune evasion (40, 60). In oral SCC, mitophagy in cancer-associated fibroblasts establishes metabolic coupling with tumor cells. This interaction further suppresses immune cell infiltration and drives tumor progression (49).

### 5.4 Commonalities and heterogeneity of mitophagy regulation across skin cancer subtypes

All three major skin cancer subtypes share several core mitophagy regulatory characteristics. First, oxidative stress induced by ultraviolet radiation acts as a common upstream trigger of mitophagy. UV-induced mitochondrial reactive oxygen species accumulation and mitochondrial DNA damage consistently activate the PINK1-Parkin pathway in all three subtypes, representing a conserved adaptive response to genotoxic stress (29). Second, hypoxic tumor microenvironment is a universal driver of receptor-mediated mitophagy. HIF-1α-dependent upregulation of BNIP3/NIX is observed in melanoma, SCC and BCC, which supports tumor cell survival under nutrient and oxygen deprivation (30, 80, 81). Third, mitophagy in all subtypes exerts a dual functional context-dependency. Moderate protective mitophagy sustains mitochondrial homeostasis and promotes tumor survival and drug resistance, whereas excessive lethal mitophagy triggers mitochondrial depletion and tumor cell death.

Despite these commonalities, the dominant mitophagy pathways, driving factors and functional consequences differ substantially across subtypes. Melanoma exhibits the most complex mitophagy profile with concurrent activation of both ubiquitin-dependent and ubiquitin-independent pathways. BRAF-mutant melanoma predominantly relies on the Parkin pathway for maintaining redox homeostasis and driving M2 macrophage polarization, whereas NRAS-mutant melanoma mainly depends on the BNIP3 pathway for metabolic reprogramming and metastatic progression (82, 83). The unique UV-microphthalmia-associated transcription factor (MITF) regulatory axis endows melanoma with a mitophagy-PD-L1 bidirectional regulatory loop that is absent in other skin cancer subtypes (84, 85). Basal cell carcinoma shows a moderate mitophagy activation pattern dominated by the PINK1-Parkin pathway. Unlike melanoma, BCC mitophagy is mainly driven by chronic ultraviolet exposure rather than oncogenic mutations. A unique synergistic interaction exists between mitophagy and the Hedgehog signaling pathway (86). Squamous cell carcinoma displays prominent spatial heterogeneity of mitophagy. The hypoxic tumor core exhibits excessive mitophagy activation mainly via the BNIP3 pathway, whereas peripheral tumor regions show impaired mitophagy and local inflammatory responses. HPV-associated SCC has a unique mitophagy profile. HPV E7 oncoprotein degrades AMBRA1 to suppress bulk autophagy (87).

## 6 Context-dependent therapeutic strategies targeting mitophagy in skin cancer

### 6.1 Mitophagy inhibition strategies for advanced melanoma as an immune-hot tumor

Advanced melanoma is classified as an immune-hot tumor. The core therapeutic strategy relies on the combination of mitophagy inhibitors with immune checkpoint inhibitors and targeted agents (88, 89). Preclinical studies have confirmed that blocking the core PINK1-Parkin and BNIP3 pathways can restore the infiltration and cytotoxic function of CD8^+^ T cells. This intervention also enhances the therapeutic efficacy of immune checkpoint inhibitors (48, 90). Natural compounds including tetrandrine and arctigenin can target key mitophagy mediators. They induce lethal mitophagy and improve the sensitivity of tumor cells to chemotherapeutic drugs (88, 89). Sonodynamic therapy combined with mitophagy inhibitors can trigger mitochondrial damage and immunogenic cell death. This combination further strengthens anti-tumor immune responses (91). Current investigations of this strategy are mainly based on cell experiments and animal models. Some natural compounds have shown verified anti-tumor effects *in vivo*. However, they have not yet entered the stage of large-scale clinical trials.

### 6.2 Mitophagy modulation combined with immunosensitization strategies for basal cell carcinoma as an immune-cold tumor

BCC acts as an immune-cold tumor. The core therapeutic regimen combines mitophagy regulators with immunosensitizing agents and Hedgehog pathway inhibitors (92, 93). Low-dose mitophagy activators can induce moderate mitophagy. They trigger immunogenic cell death and convert immune-cold tumors into immune-hot phenotypes (89). Imiquimod, a TLR7 agonist, can activate local cutaneous immunity by inducing mitophagy. Its combination with Hedgehog inhibitors helps break the immunosuppressive state (68, 94). Nanogel-based local delivery systems allow high-concentration drug enrichment in cutaneous lesions. These systems reduce systemic adverse effects and improve the efficacy of local immunosensitization (95, 96). This strategy is mainly investigated in animal models and *in vitro* organoid experiments. Nanomedicines and natural small molecules represent the core intervention tools. The whole strategy is still at the preclinical research stage (95).

### 6.3 Precise mitophagy targeting combined with chemotherapy for squamous cell carcinoma as a moderately immune-infiltrated tumor

Oral and cutaneous SCC belong to tumors with moderate immune infiltration. The core therapeutic strategy relies on precise mitophagy targeting combined with chemotherapy and immune checkpoint inhibitors (97–99). Inhibitors targeting molecules including Mcl-1 and SIRT5 can induce lethal mitophagy. They enhance the cytotoxicity of chemotherapeutic agents and reverse chemoresistance (100, 101). Mitochondria-targeted nanocarriers can specifically accumulate in hypoxic regions of tumors. They inhibit overactivated mitophagy and improve the response rate to immune checkpoint inhibitors at the same time (95, 96). Analyses of clinical samples have confirmed that mitophagy-related genes can be used as predictive biomarkers for the response of SCC to chemotherapy and immunotherapy. These biomarkers can further guide stratified treatment for patients (69, 102). This strategy has been validated in *in vitro* cell assays, animal models and clinical sample analyses. Several agents such as dihydroartemisinin have entered the phase of small-scale clinical investigation (98, 103).

### 6.4 Mechanisms of therapeutic resistance and corresponding solutions

Resistance mechanisms underlying mitophagy-targeted therapy in skin cancer mainly include tumor metabolic compensation, remodeling of mitochondrial function, and adaptive changes in the tumor microenvironment (66, 104, 105). Skin cancer cells resistant to targeted drugs and chemotherapeutic agents maintain cell survival by upregulating protective mitophagy. Resistance to immunotherapy is associated with mitophagy-mediated functional abnormalities in immune cells (8, 104). Glycolysis inhibitors can be combined with fatty acid synthase inhibitors to target metabolic compensation. This combination blocks compensatory metabolic pathways in tumor cells (49, 106). Mitochondrial dynamics modulators can be used to regulate the expression of DRP1 and MFN2. This intervention counteracts mitochondrial remodeling and restores normal mitochondrial function (107, 108). Synthetic lethal strategies have become a new direction for overcoming drug resistance. The coexistence of NF1 deletion and defective lysosomal trafficking can specifically eliminate drug-resistant melanoma cells by blocking mitophagy (109).

## 7 Challenges and future perspectives

### 7.1 Core challenges

Marked heterogeneity exists in mitophagy function across different subtypes of skin cancer. A single intervention strategy cannot be applied to all subtypes, and subtype-specific targeted regimens remain lacking (14, 110). Mitophagy is highly context-dependent. The transition boundary between protective mitophagy and lethal mitophagy is poorly defined. Inappropriate intervention may easily lead to opposite therapeutic outcomes. In the skin cancer microenvironment, mitophagy regulation in tumor cells and immune cells is intricately interconnected. Precise technologies that specifically target mitophagy in tumor cells are currently unavailable. Critical bottlenecks hinder clinical translation. Most interventions are still limited to cellular and animal studies. Specific clinical biomarkers for monitoring mitophagy activity have not been established (69, 102). Local targeted formulations suffer from insufficient delivery efficiency and stability. Poor transdermal absorption makes it difficult to achieve effective drug concentrations in lesion tissues.

### 7.2 Future research directions

Future studies should deepen investigations into skin cancer specific regulatory mechanisms. The regulatory network of mitophagy in Langerhans cells needs to be elucidated. The complete signaling axis involving ultraviolet radiation, MITF, mitophagy and epigenetic modifications should also be clearly defined. Single cell multi omics and spatial metabolomics can be employed to construct heterogeneity maps of mitophagy across different skin cancer subtypes and tumor regions. This will facilitate precise tumor classification (19, 69, 102). Topical skin-targeted mitophagy modulators should be developed. Transdermal nanogels and microneedle delivery systems are promising platforms to improve local drug concentrations in lesions and reduce systemic toxicity (96). Liquid biopsy biomarkers based on mitophagy gene signatures should be established. These tools will enable noninvasive prediction and dynamic monitoring of immunotherapy responses in skin cancer. Innovative combination therapeutic strategies warrant exploration. The synergistic effects of mitophagy modulators with personalized cancer vaccines and oncolytic viruses can be investigated to further enhance the efficacy of immunotherapy.

## 8 Conclusion

Mitophagy serves as a central hub regulating immune evasion in skin cancer. It drives the formation and stabilization of the immunosuppressive tumor microenvironment in skin cancer through three major dimensions, including the reprogramming of immune cell functions, the coupling between metabolic reprogramming and immunosuppression, and epigenetic modifications. A bidirectional regulatory relationship exists between mitophagy and PD-L1. This regulatory network is characterized by the skin-cancer-specific ultraviolet radiation-MITF axis.

Distinct heterogeneity is observed in immune phenotypes and mitophagy profiles among different skin cancer subtypes. Melanoma is featured by excessive mitophagy activation, BCC by moderate mitophagy activation, and SCC by spatial heterogeneity in mitophagy activity. Context-dependent therapeutic strategies based on immune phenotypes represent a key approach to overcoming immunotherapy resistance in skin cancer. Mitophagy inhibition is recommended for immune-hot tumors, mitophagy modulation combined with immunosensitization for immune-cold tumors, and precise mitophagy targeting combined with chemotherapy for moderately immune-infiltrated tumors.

Current research in this field still faces challenges such as heterogeneous regulation, targeted delivery, and clinical translation. Future multidisciplinary studies are required to deepen mechanistic insights, innovate technical tools, and optimize therapeutic regimens. These efforts will promote the translation of mitophagy-targeted strategies from basic research to clinical application, providing more precise and effective therapeutic options for patients with skin cancer.
